# Design, synthesis and biological evaluation of N-substituted α-hydroxyimides and 1,2,3-oxathiazolidine-4-one-2,2-dioxides with anticonvulsant activity

**DOI:** 10.1080/14756366.2019.1651722

**Published:** 2019-08-14

**Authors:** Laureano L. Sabatier, Pablo H. Palestro, Andrea V. Enrique, Valentina Pastore, María L. Sbaraglini, Pedro Martín, Luciana Gavernet

**Affiliations:** a Medicinal Chemistry, Department of Biological Sciences, Faculty of Exact Sciences, National University of La Plata, La Plata, Argentina;; b Instituto de Estudios Inmunológicos y Fisiopatológicos (IIFP), CONICET—Universidad Nacional de la Plata), Facultad de Ciencias Exactas, Universidad Nacional de La Plata, La Plata, Argentina

**Keywords:** Epilepsy, 1,2,3-oxathiazolidine-4-one-2,2-dioxide, α-hydroxyimides, docking, sodium channels blockers, MES test, microwave-assisted synthesis

## Abstract

In this investigation, we studied a family of compounds with an oxathiazolidine-4-one-2,2-dioxide skeleton and their amide synthetic precursors as new anticonvulsant drugs. The cyclic structures were synthesized using a three-step protocol that include solvent-free reactions and microwave-assisted heating. The compounds were tested *in vivo* through maximal electroshock seizure test in mice. All the structures showed activity at the lower doses tested (30 mg/Kg) and no signs of neurotoxicity were detected. Compound encoded as **1g** displayed strong anticonvulsant effects in comparison with known anticonvulsants (ED^50^ = 29 mg/Kg). First approximations about the mechanisms of action of the cyclic structures were proposed by docking simulations and *in vitro* assays against sodium channels (patch clamp methods).

## Introduction

**Figure 1.**
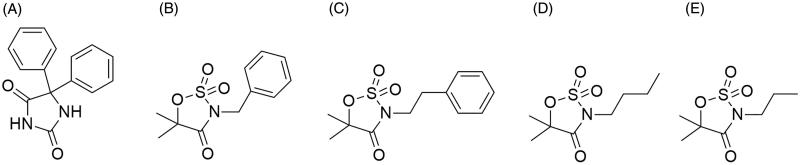
Chemical structure of phenytoin (A), and the oxathiazolidine-4-one-2,2-dioxides previously synthesized (B–E).^5^

The advances in the understanding of epilepsy and its comorbid conditions have triggered notable improvements of the pharmacological options for the treatment of this disorder. The new developments include innovative processes like the use of 3D printing technologies for improving the delivery of anticonvulsant drugs (ACD)^1^, but mostly involve the introduction of new approved compounds^2^. There are more than 20 ACDs currently available for use in the United States, which means that the therapeutic arsenal has been doubled over the past 15 years^2^. However, the discovery of more effective compounds continues to be a top priority for researches in the field^3^. According to World Health Organization, there are 50 million people worldwide with epilepsy and one-third of them are unable to control the disorder with ACDs^4^. Therefore, most of the efforts are now focused on lowering the number of patients that experience resistance to the available medications. In this investigation, we studied a family of structures with an oxathiazolidine-4-one-2,2-dioxide skeleton and their synthetic precursors as new candidates of ACDs. The rationale for selecting the cyclic scaffold was based on its bioisosteric relationship with the classical ACD phenytoin (Figure 1). The anticonvulsant action of phenytoin was identified by Putnam and Merrit in 1937 by means of their pioneering model for rapid screening of ACD, which tested the ability of candidates to protect against electroshock-induced convulsions in cats^6^. This phenotypic screening was the starting point for the development of acute models of seizures in animals that successfully identified the majority of known ACDs^7^. Even today phenytoin is used in epilepsy treatment for generalized tonic-clonic and partial seizures^8^.

In a previous work, on some N-derivatives of oxathiazolidine-4-one-2,2-dioxides, we synthesized only four new compounds with the scaffold and they showed activity against the Maximal Electroshock Seizure (MES) test in mice (Figure 1)^5^. The MES test consist of the electrical induction of the convulsions and it is associated with the generalized tonic–clonic seizures^9^. Interestingly, the synthetic precursors of the cycles, α-hydroxyamides, also exhibited anticonvulsant action in MES test^5^. These results encouraged us to expand the set with new structures, with the aim of exploring the anticonvulsant action in both α-hydroxyamides and their cyclic derivatives.

The synthetic procedure involved the preparation of the α-hydroxyamides from the ammonolysis reaction between 2-hydroxyisobutyl methyl ester and the corresponding amine followed by a cyclization reaction and the oxidation of the product^5^. Both sets of α-hidroxyamides and oxathiazolidine-4-one-2,2-dioxide derivatives were evaluated against MES test in mice^9^. Toxicity was also tested by the standardized Rotorod test, which is also included in the primary phase of anticonvulsant screening program^9^. To get inside into the possible mode of action of the compounds, we analysed the capacity of the structures of blocking the Nav1.2 isoform of the voltage-gated sodium channels (VGSC). The selection of the target was supported on preliminary studies about one compound of the cyclic family, the 3-butyl-5,5-dimethyl oxathiazolidine-4-one-2,2-dioxide (compound D, Figure 1), which has sodium channels (VGSC) blocking properties^10^. Additionally, it is well known that Nav1.2 isoform is the molecular target of many ACDs like phenytoin, lamotrigine, carbamazepine, oxcarbazepine, eslicarbazepine, zonisamide and lacosamide^11^^,^^12^. First, we simulated the interaction between the compounds synthesized and a 3D model of the Nav1.2 isoform by means of docking protocols. Then, the docking candidates and other structures predicted as inactive were tested on Nav1.2 currents using the patch clamp technique.

## Materials and methods

### Chemistry

Microwave reactions were carried out in an Anton-Paar-monowave-300 reactor (monowave, maximum power 850 W, temperature control via IR-sensor, vial volume: 10–30 mL). Melting points were determined using capillary tubes with an electrothermal melting point apparatus and are uncorrected. Thin-layer chromatography (TLC) was performed with aluminium backed sheets with silica gel 60 F254 (Merck, ref 1.05554), and the spots were visualized with 254 nm UV light and 5% aqueous solution of ammonium molybdate (VI) tetrahydrate. Column chromatography was performed on silica gel 60 (70–230 mesh, Merck, ref 1.07734.2500). ^1^H and 13C NMR spectra were recorded on a Bruker Avance 500 MHz spectrometer. The chemical shifts were reported in ppm (δ scale) relative to internal TMS, and coupling constants were reported in Hertz (Hz).

*Synthesis of α-hydroxyamides 1(a*–*g).* Compounds **1d** and **1e** were previously synthesized^5^^,^^13–17^. MW-assisted free solvent synthesis was performed by means of the previously developed procedures:^5^ α-hydroxyisobutylmethyl ester (20 mmol) was put into a dry vessel with the corresponding amine (24 mmol) and a Teflon-coated magnetic stirring bar. The reactor was set at 150 °C, the reaction time was 30 min and the mixtures were monitored by TLC. Amides **1b**, **1d** and **1f**, were also synthesized by conventional heating and 30% w/w of 1,5,7-triazabicyclo [4.4.0] dec-5-ene (TBD) as catalyst to improve the yield of the products achieved with MW heating. These reactions were conducted at 70 °C during 20 h of reaction and the yields with this alternative route increased from 24 to 32% for **1b**, 5 to 56% for **1d** and 18 to 47% for **1f**.

The isolation steps were similar in both conventional and microwave assisted heating. After the reaction was concluded, dichloromethane (30 mL) was added and the solution was washed with 10 mL of 5% v/v hydrochloric acid (2×) and brine (1×). The combined organic layers were dried over Na_2_SO_4_ and concentrated under reduced pressure. The residue was then purified by silica column chromatography and/or crystallization. The pure products were obtained as white solids in all cases except for compound **1a**.

**N-hexyl-2-hydroxyisobutylamide (1a)**. Yield 40% (oil). ^1^H NMR (500 MHz, CDCl_3_) δ 6.75 (*s*, 1H, –N**H**), 3.26 (*m*, 2H, –C**H_2_**–NH), 1.53 (*m*, 2H, –C**H_2_**–CH_2_–NH–), 1.47 (*s*, 6H, –C**H_2_**–), 1.32 (*s*, 6H, (–C**H_3_**)_2_), 0.90 (*t*, *J = 6.7 Hz*, 3H, –C**H_3_**). 13C NMR (126 MHz, CDCl_3_) δ 175.91 (**C**O), 73.29 (–**C**–OH), 39.48 (–**C**H_2_–NH–), 31.16 (–**C**H_2_–(CH_2_)_3_–NH–), 29.51(–**C**H_2_–CH_2_–NH–), 27.61 ((–**C**H_3_)_2_), 26.54 (–**C**H_2_–(CH_2_)_2_–NH–), 22.76 (–**C**H_2_–(CH_2_)_4_–NH–), 13.8 (–**C**H_3_).

**N-isopropyl-2-hydroxyisopropylamide (1b)**. Yield with MW heating: 24%, yield with conventional heating 32% (mp: 66–67, hexane). ^1^H NMR (500 MHz, CDCl_3_) δ 6.71 (*s*, 1H, –N**H**), 4.05–3.88 (*m*, 1H, –C**H**), 3.40 (*s*, 1H, –O**H**), 1.43 (*s*, 6H, (–C**H_3_**)_2_), 1.16 (d, *J = 6.6 Hz*, 6H, –CH–(C**H_3_**)_2_). 13C NMR (126 MHz, CDCl_3_) δ 175.96 (**C**O), 72.52 (–**C**–OH), 40.64 (–NH–**C**H–), 27.54((–**C**H_3_)_2_), 22.24(–CH–(**C**H_3_)_2_).

**N-isobutyl-2-hydroxyisobutylamide (1c)**. Yield 57% (mp: 104–105, hexane). ^1^H NMR (500 MHz, CDCl_3_) δ 6.17 (*s*, 1H, –N**H**), 3.08 (*t*, *J = 6.5 Hz*, 2H, –C**H_2_**), 1.84–1.71 (*m*, 1H, –C**H**), 1.45 (*s*, 6H, (–C**H_3_**)_2_), 0.93 (d, *J = 6.7 Hz*, 6H, –CH–(C**H_3_**)_2_) 13C NMR (126 MHz, CDCl_3_) δ 176.23 (**C**O), 73.33(–**C**–OH), 46.37(–NH–**C**H_2_–), 28.55(–NH–CH_2_–**C**H–), 27.95 ((–**C**H_3_)_2_), 20.02(–CH–(**C**H_3_)_2_).

**N-(1-ethyl) benzyl-2-hydroxyisobutylamide (**1f**)**. Yield with MW heating: 18%, Yield with conventional heating: 47% (mp: 63–64, hexane). ^1^H NMR (500 MHz, CDCl_3_) δ 7.38–7.27 (*m*, 5H, Ar), 7.02 (1, 1H, –N**H**), 4.86 (*q*, 1H, –NH–C**H**–Ar), 2.13 (*s*, 1H, –O**H**), 1.86 (*p*, *J = 7.3 Hz*, 2H, –C**H_2_**–), 1.49 (*s*, 3H, –C**H_3_**), 1.44 (*s*, 3H, –C**H_3_**), 0.93 (*t*, *J = 7.4 Hz*, 3H, –CH_2_–C**H_3_**). 13C NMR (126 MHz, CDCl_3_) δ 175.50 (**C**O), 142.15 – 126.49 (Ar), 73.68 (–**C**–OH), 54.54 (–NH–**C**H–), 29.38–28.06 ((–**C**H_3_)_2_), 27.86 (–**C**H_2_–CH_3_),10.69 (–CH_2_–**C**H_3_).

**N-(p-fluoro) benzyl-2-hydroxyisobutylamide (**1g**).** Yield 28% (mp: 73–74.5, hexane). ^1^H NMR (500 MHz, CDCl_3_) δ 7.27–6.97 (*m*, 5H, Ar + NH), 4.41(d, *J = 6.0 Hz*, 2H, –NH–C**H_2_**–Ar), 2.22 (*s*, 1H, –O**H**), 1.48 (*s*, 6H, (–C**H_3_**)_2_). 13C NMR (126 MHz, CDCl_3_) δ 176.02 (**C**O), 163.16, 161.20 (**C**–F), 134.07 –115.48 (**Ar**), 73.78 (–**C**–OH), 42.57(–NH–**C**H_2_–), 28.04 ((–**C**H_3_)_2_).

*Synthesis of N-derivative-1,2,3-oxathiazolidine-4-one-2,2-dioxides 3(a, c-e, g).* First, we synthesized the monoxides as previously described for cyclic sulfamates^5^. To a mixture of N-substituted amide-2-hydroxyisobutylamide (3 mmol) and triethylamine (13 mmol) in CH_2_Cl_2_ anhydrous (8 mL) at 0 °C, thionyl chloride (4 mmol) in CH_2_Cl_2_ anhydrous (4 mL) was added dropwise. The mixture was stirred under N_2_ overnight and concentrated to dryness under reduced pressure to give N-derivative-1,2,3 oxatiazolidine-4-one-2-oxides as yellow oils. Then, the dioxides were obtained after oxidation with NaIO_4_ and RuCl_3_.6H2O:^18–20^ An aqueous solution of NaIO_4_ (7 mmol) and ruthenium chloride was added dropwise to a solution of N-derivative-1,2,3-oxathiazolidine-4-one-2-oxides (4.5 mmol) in acetonitrile (6 mL) and dichloromethane (6 mL) at 0 °C. The mixture was warmed to room temperature and an hour later extracted twice with CH_2_Cl_2_. The organic phases were combined, washed with water and brine, dried (Na_2_SO_4_) and concentrated to dryness. The residue obtained was flash chromatography on silica-gel 60 (70–230 mesh, Merck) and mixtures of CH_2_Cl_2_/hexane to give a white solid for all the compounds excluding **3a**.

**3-hexyl-5,5-dimethyl-1,2,3-oxathiazolidine-4-one-2,2-dioxide (3a)**. Yield 25% (oil). ^1^H NMR (500 MHz, CDCl_3_) δ 3.67 (*m*, 2H, –CH_2_–N), 1.79 (*m*, 2H, –C**H_2_**), 1.76 (*s*, 6H, (–C**H_3_**)_2_), 1.34–1.29 (*m*, 6H, –C**H_2_**), 0.91 (*t*, *J = 6.8 Hz*, 3H, –C**H_3_**). 13C NMR (126 MHz, CDCl_3_) δ 168.38 (**C**O), 93.26 ((CH_3_)_2_–**C**–O–), 42.03 (–N–**C**H–), 30.98 (–**C**H_2_–CH_2_–N–), 27.46 (–**C**H_2_–(CH_2_)_3_–N–), 26.09 (–**C**H_2_–(CH_2_)_2_–N–), 24.35 (–**C**H_2_–(CH_2_)_4_–N–), 22.49 ((–**C**H_3_)_2_), 13.85 (–**C**H_3_).

**3–(1-isobutyl) 5,5-dimethyl-1,2,3-oxathiazolidine-4-one-2,2-dioxide (3c)**. Yield 55% (mp: 40–41, hexane). ^1^H NMR (500 MHz, CDCl_3_) δ 3.49 (d, *J = 7.7 Hz*, 2H, –N–C**H_2_**–), 2.30 – 2.19 (*m*, 1H, –C**H**–CH_3_), 1.77 (*s*, 6H,(–C**H_3_**)_2_), 1.00 (d, *J = 6.7 Hz*, 6H, –CH–C**H_3_**). 13C NMR (126 MHz, CDCl_3_) δ 168.90 (**C**O), 93.21 ((CH_3_)_2_–**C**–O–), 48.99 (–N–**C**H_2_–), 27.13 (–N–CH_2_–**C**H–), 24.44 ((–**C**H_3_)_2_), 19.72 (–N–CH_2_–CH–(**C**H_3_)_2_).

**3-cyclohexyl-5,5-dimethyl-1,2,3-oxathiazolidine-4-one-2,2-dioxide (3d).** Yield 71% (mp: 59–60, hexane). ^1^H NMR (500 MHz, CDCl_3_) δ 4.00–3.92 (*m*, 1H, –C**H**–), 2.06 – 1.17 (*m*, 10 H, cyclohexyl), 1.73 (*s*, 6H, (–C**H_3_**)_2_) 13C NMR (126 MHz, CDCl_3_) δ 168.46 (**C**O), 91.81 ((CH_3_)_2_–**C**–O–), 56.05 (–N–**C**H–), 29.77, 25.71, 24.82 (cyclohexyl), 24.28 ((–**C**H_3_)_2_).

**3–(1-methyl) benzyl-5,5-dimethyl-1,2,3-oxathiazolidine-4-one-2,2 dioxide (3e).** Yield 58% (mp:66–67, hexane). ^1^H NMR (500 MHz, CDCl_3_) δ 7.53 – 7.27 (*m*, 5H, Ar), 5.32 (*q*, 1H, –C**H**), 1.94 (d, *J = 7.3 Hz*, 3H, CH–C**H_3_**), 1.81 (*s*, 3H, –C**H_3_**), 1.51 (*s*, 3H, –C**H_3_**) 13C NMR (126 MHz, CDCl_3_) δ 163.06 (**C**O), 133.81–115.44 (Ar), 73.24 ((CH_3_)_2_–**C**–O–), 41.68 (–N–**C**H–), 27.55 ((–**C**H_3_)_2_), 27.20 (–CH–**C**H_3_).

**3-p-fluorbenzyl-5,5-dimethyl-1,2,3-oxathiazolidine-4-one-2,2-dioxide (3g)**. Yield 52% (mp: 86–87, dichloromethane/hexane). ^1^H NMR (500 MHz, CDCl_3_) δ 7.47 – 7.39 (*m*, 2H, Ar), 7.08 (*m*, 2H, Ar), 4.77 (*s*, 2H, –C**H_2_**–Ar), 1.76 (*s*, 6H, (–C**H_3_**)_2_). 13C NMR (126 MHz, CDCl_3_) δ 168.44 (**C**O), 163.89, 161.92 (**C**–F), 130.87 –115.82 (Ar), 93.56 ((CH_3_)_2_–**C**–O–), 44.72 (–N–**C**H_2_–), 24.30 ((–**C**H_3_)_2_).

### Biological assays

*In vivo test.* We used male albino mice (18–23 g) provided by the Faculty of Veterinary, of the National University of La Plata. They were maintained under a regime of 12-h light/dark cycle and allowed free access to food and water. The animal care for the experimental protocols was conducted in accordance with the National Institutes of Health (NIH) guidelines for the Care and Use of Laboratory Animals and it was approved by the Ethical Committee of Exact Sciences Faculty of University of La Plata. Mice were randomized to different treatments. Candidate’s solutions were performed in polyethyleneglycol 400 (PEG 400) at a rate of 3 mL/kg body weight and physiological solution was added up to a maximum volume of 7 mL/kg. Mice were i. p. administered with the synthetized compounds at doses of 30 or 100 mg/kg and they were evaluated at 0.5 or 4 h.

Maximal electroshock seizures were provoked in mice by delivering a 60 Hz/50 mA electrical stimulus for 0.2 s via ear clip electrodes by means of a UGO Basile equipment. In these conditions, normal mice experience maximal seizures, characterized by a short period of tonic flexion followed by a longer period of tonic extension of the hind limbs and a final clonic episode^9^. Three minutes before induction of convulsion, all animals were evaluated in the Rotorod test. Rotorod equipment consists of a fluted roll divided by opaque discs rotating at a speed of 6 rpm. Animals were arranged on the cylinder and the ability to maintain balance on the rotarod for 1 min, in three consecutive tests, was evaluated. The inability of animals to maintain balance during the three tests, showing ataxia and sedation, was considered a sign of neurotoxicity^21^. Quantitative studies in MES test were conducted for **1g** at the previously determined time of peak effect (TPE). The ED^50^ was determined by treating groups of six albino mice. Different doses were used for each drug at TPE. The method of Litchfield and Wilcoxon was used to compute the ED50 value^22^.

*Electrophysiology.* The electrophysiological recordings were performed using the patch-clamp technique in HEK293 cell line stably expressing the hNav1.2 channel isoform (a kind gift from GlaxoSmithKline, Stevenage, UK). The standard tight-seal whole-cell configurations of the patch-clamp technique was used to record macroscopic currents^23^. Whole-cell currents were filtered with a 4-pole lowpass Bessel filter (Axopatch 200 A amplifier) at 2 kHz and digitized (Digidata 1440, Molecular devices) at a sample frequency of 200 kHz (5 µs). Once the whole-cell configuration was obtained, current stability was evaluated with a 15 ms-voltage-clamp step from a holding potential of −80 mV to a test potential of −20 mV repeated each 10 s. The time needed for the stabilization was variable (approximately 10 min). The same voltage-clamp step protocol was applied in the control (vehicle) or in the presence of compounds **1e**, **1g**, **3e** and **3g**, dissolved in 0.1% dimethylsulphoxide. After current stabilization on each condition, the voltage dependence of the steady-state inactivation of sodium channels was evaluated using a double voltage step protocol, where the same depolarization to −10 mV followed different pre-conditioning 2.5 s steps (from −130 to −40 mV). The available fraction of sodium channels at each membrane potential (IVc/Imax) was calculated as the ratio of peak sodium current measured at −10 mV, at each pre-conditioning voltage test pulse (IVc) and the maximum peak current observed (Imax). The relationship between the available fraction of sodium channels and the pre-conditioning (named h curve) was plotted and fitted with a Boltzmann equation (Equation (1)):

(1) IVc/Imax=1/(1+exp((Vh−V)/k))

where the available fraction is given as IVc/Imax, Vh is the potential of half-maximal inactivation and k is the slope parameter. Statistical significance of the changes in the Vh parameter induced by the compounds was tested with F method (GraphPad Prism). More details of the experiment are given in Supporting online material.

*Docking simulations.* The molecules were docked into the model of the 3D structure of the α-subunit of the human Nav1.2 (open-pore conformation) previously constructed by us^24^. The “docking active site” was defined based on the experimental data, and includes residues numbered from Phe1764 to Tyr1771 as important for the interactions of the channel with known ACDs^25^. Previous analyses of the performance of different docking protocols allowed us to select Autodock Vina^26^ as the best method to discriminate known binders from non-binders of Nav1.2 through the docking score, so the conditions of the docking are already reported. We set the cut-off value as −8.1 Kcal/mol to differentiate active from inactive compounds, which has associated specificity and sensibility rates of 80% and 88%, respectively, in the receiver operating characteristic (ROC) curve of the set of compounds used for the validation of the protocol^24^.

## Results and discussion

### Chemistry

**Scheme 1.**
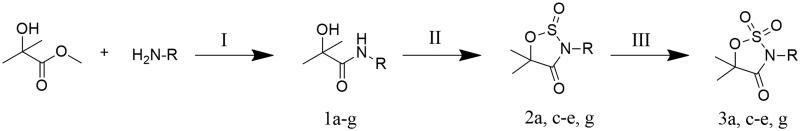
Synthetic route used in this investigation: (I) Ammonolysis reaction: MW radiation or catalyst, N_2_ atmosphere, (II) Cyclization reaction: SOCl_2_, triethylamine, (III) Oxidation reaction: NaIO_4_ RuCl_3_.6H_2_O. Code for compounds: (1) α-hydroxyamide, (2) N-derivative-1,2,3-oxathiazolidine-4-one-2-oxide, (3) N-derivative-1,2,3-oxathiazolidine-4-one-2,2—dioxide. And a = hexyl, b = isopropyl, c = isobutyl, d = cyclohexyl, e = 1-methylbenzyl, f = 1-ethylbenzyl, g = p-fluorbenzyl.

The synthetic route followed in this investigation is shown in Scheme 1.

*Synthesis of N-substituted 2-hydroxyisobutyl methyl amides*. The α-hydroxyamides 1(a–g) were synthesized by ammonolysis of 2-hydroxyisobutyl methyl ester (Scheme 1). Compounds **1a**, **1c**, **1e** and **1g** were achieved under a solvent-free environment and microwave (MW)-assisted heating^5^. In these conditions, compounds **1b**, **1d** and **1f** were obtained with low yields. To improve the synthetic method for these amides, we conducted the reactions with a conventional heating system, and we used TBD as catalyst. The selection of this compound was supported by previous studies about the synthesis of other secondary and tertiary amides under solvent-free conditions^27^.

*Synthesis of N-derivatives-5,5-dimethyl oxathiazolidine-4-one-2,2-dioxides.* The cyclization reaction of the resulting α-hydroxyamides to get the corresponding sulphamates was carried out following the conditions previously described for cyclic sulphamates (Scheme 1)^5^^,^^18–20^. Initially we obtained the cyclic monoxides through the reaction of amides with thionyl chloride and triethylamine. Then, the compounds were oxidized with NaIO_4_ and RuCl_3_ to yield the final dioxide derivatives. N-isopropyl (**2b** and **3b**) and (1-ethyl) benzyl (**2f** and **3f**) derivatives were not obtained in these conditions, so they were not evaluated. The yields of the reactions are in the range between 25% and 71% for pure compounds.

*In vivo tests.* We followed the standard procedures proposed by the Epilepsy Therapy Screening Program (ETSP) of the NIH^9^, which is described in the Materials and Methods section. In Table 1, we report the results of MES test after the administration of the compounds in terms of the fraction of animals that did not show hind limbs tonic extension for each dose/time group. The primary toxicity of the drugs (signs of sedation and/or ataxia) was measured by the standardized Rotorod test, also included in the primary phase of the ETSP^9^.

**Table 1.** Pharmacological profile (phase I) of the synthesized compounds.

Compound	Dose (mg/Kg)	MES^a^		Rotorod^b^		Class
**1a**	30	1/2	2/3	0/2	0/3	1
	100	2/2	2/2	0/2	0/2	
**1b**	30	0/2	3/4	0/2	0/4	1
	100	1/2	2/4	0/2	0/4	
**1c**	30	1/3	1/3	0/3	0/3	1
	100	1/3	2/3	0/3	0/3	
**1d**^(8)^	30	1/3	3/3	0/3	0/3	1
	100	3/3	2/3	0/3	0/3	
**1e**	30	0/3	1/3	0/3	0/3	1
	100	3/3	1/3	0/3	0/3	
**1f**	30	2/5	0/2	0/5	0/2	1
	100	3/5	0/2	0/5	0/2	
**1g**	30	3/4	0/2	0/4	0/2	1
	100	4/4	0/2	0/4	0/2	
**3a**	30	0/2	0/2	0/2	0/2	1
	100	2/4	2/4	0/4	0/4	
**3c**	30	0/2	0/2	0/2	0/2	1
	100	1/2	0/2	0/2	0/2	
**3d**	30	4/4	1/4	0/4	0/4	1
	100	(–)	(–)	(–)	(–)	
**3e**	30	1/2	1/2	0/2	0/2	1
	100	0/2	1/2	0/2	0/2	
**3g**	30	1/5	1/4	0/3	0/2	1
	100	0/2	0/2	0/2	0/2	

^a^Number of protected animals relative to the total number of mice tested in MES at each time and concentration. ^b^Number of animals with sedative effects relative to the total number of mice tested at each time and concentration. Compound **3d** was not tested at dose of 100 mg/kg due to solubility problems, referenced as (–). (8) See reference in Bibliographic section.

The compounds were administered to mice intraperitoneally at the lower doses of the program (30, 100 mg/kg), and all the assays were performed at 0.5 and 4 h. Table 1 includes the classification of the structures according to the following criteria^28^. Class (1): compound with anticonvulsant activity at 100 mg/kg or less, Class (2): compound with anticonvulsant activity at doses higher than 100 mg/kg, Class (3): inactive compound at any doses up to 300 mg/kg, and Class (4): inactive compound at 300 mg/kg and toxic at 30 mg/kg or less. The results achieved from the assays showed that all the structures were active against MES test, which allowed us to classify them as class 1. In fact, most of the structures exhibited protection against the induced seizure at the lower doses tested (30 mg/kg). These results are encouraging since class 1 includes the most promising candidates for the next step of the biological testing^28^. In addition to the anticonvulsant activity found for the compounds, no signs of sedation and/or ataxia were detected in the Rotorod test, which is important in terms of the safety profile of the active structures. Compound **3d** had solubility problems, so it was not possible to evaluate them at the highest dose with the recommended vehicles of the ETSP Program^6^.

ED^50^ value was calculated for compound **1g**, since it showed strong anticonvulsant action at both doses evaluated in MES test. Again, we followed the standard procedures for the calculation, described in the Materials and Methods section. Essentially, ED^50^ measures the dose of drug that is effective in 50% of the tested animals^9^. This value is calculated at the time of peak effect (TPE), which has to be previously identified^9^. The final ED^50^ value of **1g** was 29 mg/kg, which is equivalent to 0,106 mmol/kg (TPE = 0.5 h). This result is interesting in terms of potency, since it is in the range of ED^50^ values measured for classical ACDs in the same test. For example, phenytoin showed ED^50^ values of 0.0218 mmol/kg; whereas valproic acid, a representative ACD of broad spectrum, showed an ED^50^ value of 1.962 mmol/kg. Also important is the fact that all the structures passed the Rotorod test, which detects neurotoxicity in terms of sedation or ataxia; and none of the mice treated with the compounds died during the assays.

### *In silico* studies

To explore one of the possible mechanisms of action of the synthesized compounds we analyse them as sodium channel blockers. The blockage of VGSC is a validated mechanism of action of many ACD with probed clinical efficacy^7^^,^^11^^,^^12^. Among the four different VGSCs isoforms recognized in CNS (Nav1.1, Nav1.2, Nav1.3 and Nav1.6), Nav1.2 is the molecular target with confirmed interaction with many ACDs^11^^,^^12^.

Initially, the molecules were docked into the Nav1.2 channel with Autodock Vina software^26^. As the 3D structure of human Nav1.2 is not available, we employed a 3D model of this macromolecule previously constructed in our laboratory^24^. It includes the 3D architecture of the α-subunit of the Nav1.2 channel, which is functional on its own and comprises the region of interactions with ACD. A docking score of −8.1 kcal/mol was defined as the threshold value to differentiate active from inactive compounds. This cut-off value was previously selected for the screening of a virtual database, since it shows a good balance between specificity and sensibility for the test set that validated the docking model (80% and 88%, respectively)^24^. Table 2 shows the docking scores of the synthesized compounds.

**Table 2.** Docking scores of the synthesized compounds.

Compound	Score	Compound	Score
**1a**	−6.4	**1g**	−7.5
**1b**	−5.5	**3a**	−6.7
**1c**	−6.3	**3c**	−6.5
**1d**	−6.9	**3d**	−7.2
**1e**	−7.7	**3e**	−8.2
**1f**	−7.8	**3g**	−8.3

**Figure 2.**
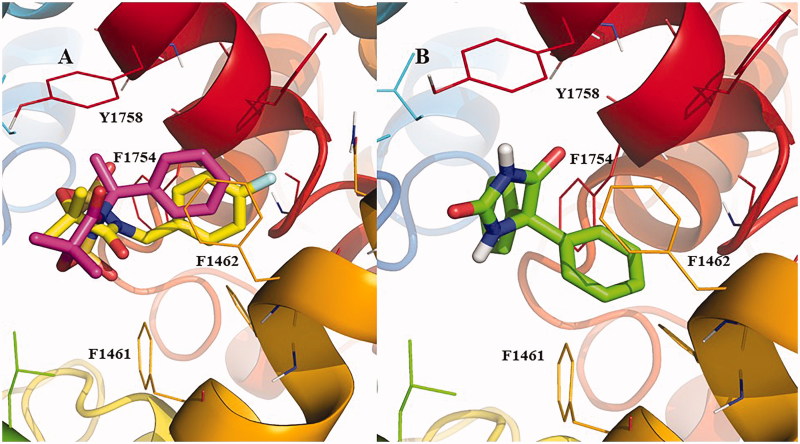
(A): Binding geometry of compounds **3e** and **3g** into the Nav1.2 active site predicted by the Autodock Vina docking algorithms. (B) binding geometry predicted for phenytoin. Carbon atoms are highlighted in pink, yellow and green for compounds **3e**, **3g** and phenytoin, respectively. Nitrogen, oxygen and fluorine atoms of the compounds are highlighted in blue, red and light blue, respectively. Residues that surround the ligand within 5 angstroms of distance are highlighted as sticks. Important aromatic residues are numbered in the figure. Hydrogen atoms are omitted for simplicity except for the non-aromatic hydrogen atoms of phenytoin.

Our docking results suggest that the amides are poorer candidates to block the Nav1.2 channel since none of them were able to pass the threshold value defined for active compounds according to the docking classificatory model (−8.1 Kcal/mol). Among cyclic structures, compounds **3e** and **3g** were predicted as active. It is worth mentioning that the docking protocol has been already validated and used to identify new Nav1.2 blockers in a previous investigation^24^. Figure 2(A) shows the binding geometries of structures **3e** and **3g** into the docking active site, which includes residues of the ion conducting pore of the channel that interact with known ACDs^25^. The simulations suggest that the compounds direct their aromatic ring towards the region delimited by aromatic side chains of Phe1462 and Phe1754. In the docking conditions, the ACD phenytoin orientates its heterocycle and one of its aromatic rings to the same regions occupied by **3e** and **3g**, with a docking score of −9.9 kcal/mmol (Figure 2(B))^26^. The overall orientation of **3e**, **3g** and phenytoin into the Nav1.2 channel is given as Supplemental online material (Figure S1).

### Electrophysiology

The virtual strategy proposed only two compounds, **3e** and **3g**, as Nav1.2 blockers. Both structures showed anticonvulsant action in mice at the lower dose tested. On the other hand, the rest of compounds (which were predicted as inactive in the Nav1.2 model) showed protection against MES test.

To analyse if the contrast between the *in silico* predictions and the *in vivo* assays can be explained as a fail in the sensibility of the docking protocol, we selected two pairs of compounds (**1e**–**3e** and **1g**–**3g**) to test their effect in Nav1.2 currents using the patch-clamp technique. The rationale for compounds selection was to test the two heterocycles predicted as active, and their corresponding α-hydroxyamides, which were classified as not active in the docking simulations (Table 2).

The *in vitro* activity of compounds **1e**, **1g**, **3e** and **3g** was evaluated in the Na^+^ currents carried through the Nav1.2 channel isoform stably expressed in HEK 293 cells by using the whole-cell configuration. The current stability, before and after perfusion of the tested compounds, was monitored every 10 s by a 10 ms voltage step to −20 mV from a holding potential of −80 mV. The inhibitory activity was observed as decay in the peak amplitude of the Na^+^ currents. After current stabilization, and with the aim to evaluate the state-dependent inhibition, we tested the ability of these compounds to modify the steady-state inactivation curves (h curves) of the Nav1.2 channels. The h curve shows the available fraction of Na + channels in condition to be activated (in resting state) as a function of the resting membrane potential (see Material and Methods section). The state-dependent inhibition, due to the stabilization of the inactivated state, is observed as a left-shift in the h curves, which means a reduction of available Na^+^ channel which is higher at more depolarized potential.

**Figure 3.**
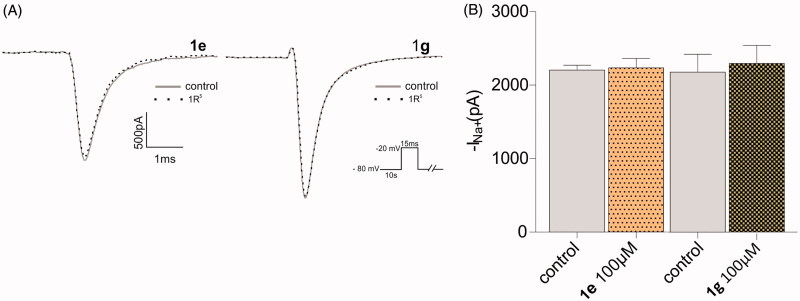
Whole cell configuration with a voltage-clamp step protocol from −80 to −20 mV. (A) Typical traces of Na + currents in Nav 1.2 isoform, control and compound **1e** or **1g**, 100 µM. (B) *p* > 0.05. No statistical differences were found for both compounds (*n* = 4).

**Figure 4.**
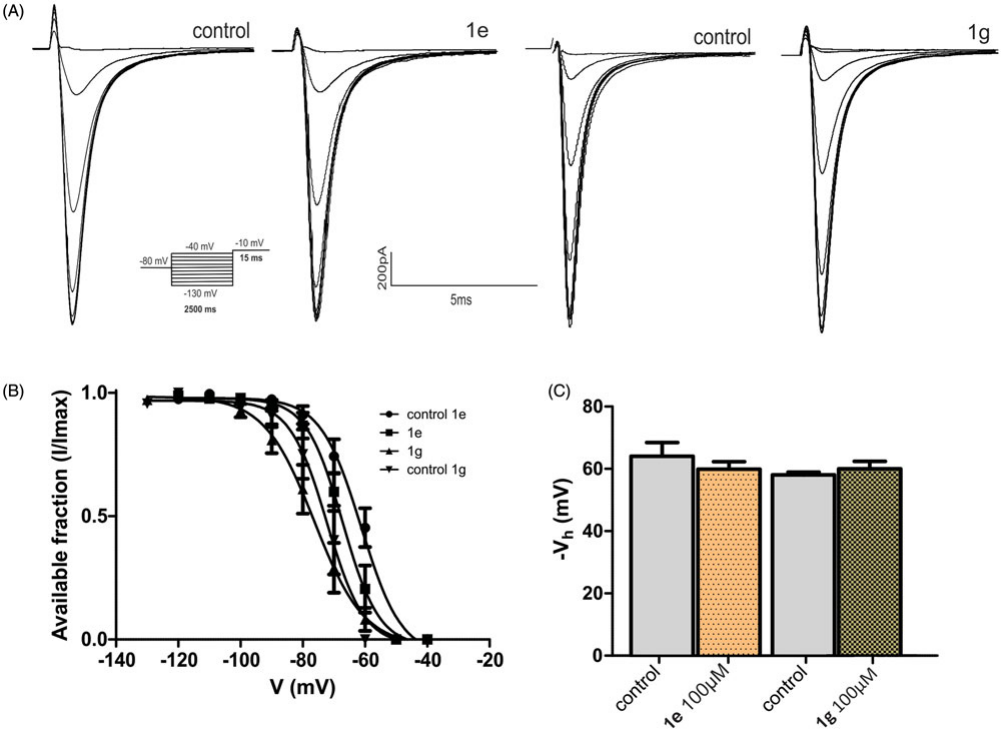
Compounds **1e** and **1g** did not affect the Nav1.2 channel steady state inactivation curves. (A) Typical traces of the steady-state inactivation protocol, where Nav1.2 currents were activated by a depolarizing voltage step to −10 mV for 15 ms following 2500 ms hyperpolarizing pre-pulses ranging from −130 to −40 mV in the absence and in the presence of 100 µ of **1e** and **1g**. (B) Mean available fraction (I/Imax) values for compound **1e** or **1g** and their respect control plotted as function of membrane voltage (mV) and fitted to the Boltzmann function. (C) Mean Vh values obtained from plots showed in (B). No statically difference was observed (*p* > 0.05, *n* = 4 for each group). Vhcontrol (**1e**) = −62.77 ± 2.5, Vh(1e) = −60.63 ± 1.40, *n* = 4. Vhcontrol (**1g**) = −58.09 ± 0.43, Vh (1 g) = −59.96 ± 1.19, *n* = 4.

**Figure 5.**
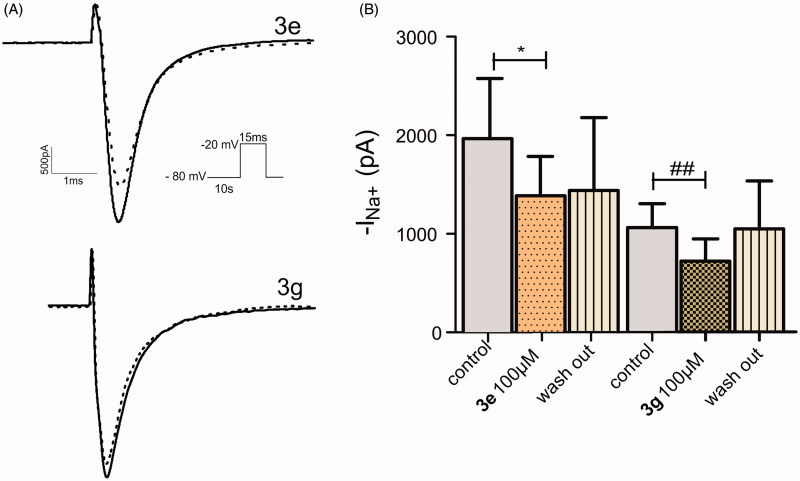
Compounds **3e** and **3g** inhibited the Nav1.2 current. (A) Typical traces of Nav1.2 currents recorded in HEK 293 cells in the whole cell configuration with a voltage-clamp step protocol from −80 to −20 mV before and after 100 µM **3e** (up) and **3g** (down) extracellular perfusion. (B) Mean Nav1.2 currents values obtained from current showed in (A). *p < 0.05 indicates significant difference between control and compound 3e (%Current Inhibition = 29.6%); ^##^*p* < 0.005 indicates significant differences between control and compound **3g** group. (%Current Inhibition = 33%). Paired *t*–test *n* = 4, for each group.

**Figure 6.**
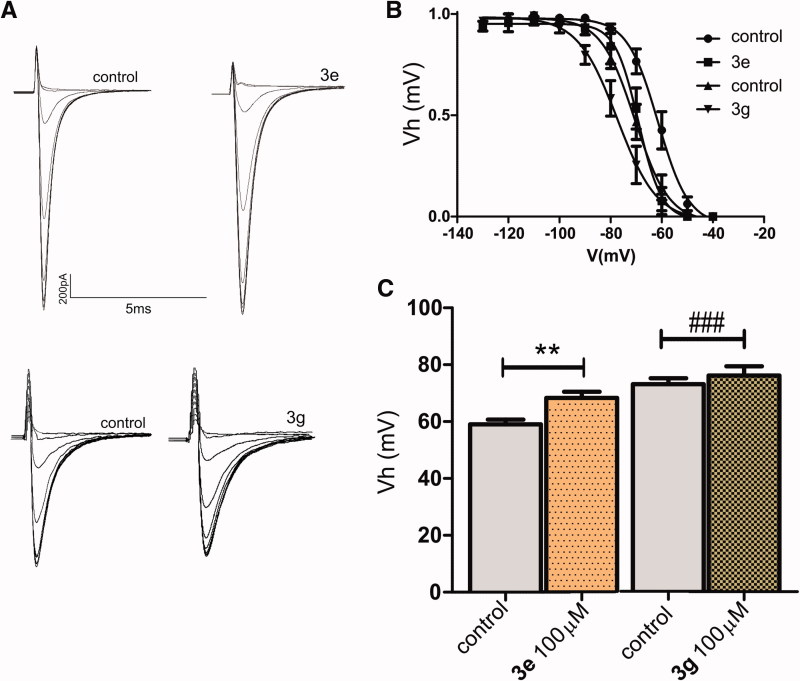
Compounds **3e** and **3g** produced a state-dependent inhibition in Nav1.2 channel. (A) Typical traces of the steady-state inactivation protocol where Nav1.2 currents were activated by a depolarizing voltage step to −10 mV for 15 ms following 2500 ms hyperpolarizing pre-pulses ranging from −130 to −40 mV in the absence and in the presence of 100 µ of **3e** and **3g**. (B) Mean available fraction (I/Imax) values for compound **3e** or **3g** and their respect control plotted as function of membrane voltage (mV) and fitted to the Boltzmann function (Equation (1)). (C) Vh values obtained from plots showed in (B). *p* < 0.05. **, ### Significant differences between **3e**, **3g** and their respective control groups, Vhcontrol (**3e**) = −60.18 ± 1.68, Vh(e) = −68.19 ± 2.19, *n* = 5, Vhcontrol (3 g) =-74.76 ± 1.26 Vh 3 g= −80.14 ± 3.08, *n* = 4.

Amides **1e** and **1g** were not able to block Na^+^ current (Figure 3(A,B)) and did not change the steady state inactivation curves (Figure 4(A,B,C)), while the corresponding cyclic compounds 3e and 3 g inhibited Na + current (Figure 5(A,B)) and shifted the h curve to more hyperpolarized potentials, (Figure 6 (A,B,C)). These results are consistent with the docking model predictions.

As mentioned before, the electrophysiological assays confirmed our docking predictions, showing that the oxathiazolidine-4-one-2,2-dioxide derivatives **3e** and **3g** inhibit Na + currents by left-shifting the steady-state inactivation curve, suggesting the interaction of these compounds with Nav1.2 channel.

## Conclusions

In this investigation, we synthesized and studied the anticonvulsant action of new α-hydroxyimides and N-derivatives-1,2,3-oxathiazolidine-4-one-2,2-dioxides, which scaffold is a bioisosteric partner of the known ACD phenytoin. The compounds were tested in mice and they evidenced a promising anticonvulsant activity as well a good safety profile at this pre-clinical stage. Particularly, structure **1g** shows an ED^50^ comparable with classical ACDs.

Docking simulations suggested the interaction of two compounds of the set with the Nav1.2 target and patch clamp experiments were performed to corroborate the *in silico* predictions. These two structures are N-aromatic derivatives of 1,2,3-oxathiazolidine-4-one-2,2-dioxides. Conversely, N-aliphatic-derivatives and all the α-hydroxyamides were classified as non-active. However, they showed anticonvulsant action in mice. The differences observed between *in vitro* (and *in silico*) assays and *in vivo* experiments can be generated by multiple factors. First, post-transcriptional modifications such as alternative splicing, channel protein phosphorylation and/or glycosylation are capable to modify the pharmacological sensitivity of ion channels. Moreover, the activity of the channel blockers can be modified by the expression of auxiliary beta subunits and co-localization with other membrane proteins in native cells^29^. A second factor is related with the selectivity of the compounds, since most ACDs can elicit the antiepileptic effect by acting in multiple targets. They are nonselective among neuronal Nav channels isoforms, and they can modify other isoforms (such Nav 1.1 and 1.6), other ions channels (such as K^+^ and Ca^2+^ channels), ionotropic receptors (like GABA-A and NMDA) and enzymes (GABA transaminase and glutamic acid decarboxylase)^7^^,^^30^. Finally, the drug metabolism can produce active metabolites which were not tested in the docking protocol and *in vitro* experiments^31^.

Our electrophysiological experiments allowed us to explore the inhibitory mechanism of action of **3e** and **3g**. Both compounds showed inhibitory activity on Nav 1.2 isoform in the two protocols tested. Interestingly, the left shift produced in the h curve indicates that these compounds can stabilize the steady-state inactivation of the channel. This state-dependent mechanism in Nav channels is shared with ACDs such as phenytoin, carbamazepine and lamotrigine, among others^32^^,^^33^. Nav channels are voltage dependent and have at least three well recognized states^34^^,^^35^. When the cell membrane potential is hyperpolarized, the channel is in the resting closed state, then when the membrane potential rise, the channel turns to an open state and subsequently fall into a not conducting inactive state. The Nav channels are not able to open from this inactivated state and hyperpolarization is needed to return the channels to the resting state. In this way, the membrane potential value determines the fraction of Nav channels that are able to open after a depolarizing stimulus and trigger an action potential. So, drugs that stabilize the inactivated state show a higher inhibitory effect in cells where the membrane is more depolarized (are voltage-dependent). This is particularly interesting since, in epilepsy, the neurons responsible for seizure are hyper-excitable and this is, in part, due to a more positive membrane potential. Thus, the Nav channel blocking effect of these drugs is greater in neurons involved in the seizure, reducing the probability of side effect induced by the channel block in normal neurons^36^. Regarding future investigations on VGSC blockers, more studies will be completed in the future for N-derivatives of 5,5-dimethyl-1,2,3-oxathiazolidine-4-one-2,2-dioxide and their synthetic precursors, to propose their possible mechanisms of action and to optimize their anticonvulsant action. Finally, the discovery of new VGSC blockers will allow us to evaluate them in other biological assays related with CNS pathologies where the VGSCs are also implicated. Good examples would be depression and anxiety, two pathologies well recognized as comorbid with epilepsy.

## Supplementary Material

Supplemental Material
