# Supplementary material for: Design, synthesis and biological evaluation of N-substituted α-hydroxyimides and 1,2,3-oxathiazolidine-4-one-2,2-dioxides with anticonvulsant activity
Source: J Enzyme Inhib Med Chem. 2019 Aug 14;34(1):1465–73. doi: 10.1080/14756366.2019.1651722 (PMC6713207; doi:10.1080/14756366.2019.1651722)
Supplement: Supplemental Material [file IENZ_A_1651722_SM8178.pdf]

Biological Assays. Electrophysiology. The electrophysiological recordings were performed using the patch-clamp technique in HEK293 cell line stably expressing the hNav1.2 channel isoform (a kind gift from GlaxoSmithKline, Stevenage, UK). HEK293 cell lines were cultured in minimum essential medium, containing 10% fetal calf serum, and 0.5% geneticin G418 sulfate. Cells were grown in a 95% O_2/5% CO_2 atmosphere at 37°C and with 95% humidity. One to two days prior to electrophysiological recordings, the cells were plated on glass coverslips. The cells were grown on glass coverslips and they were observed with a mechanically stabilized inverted microscope (Telaval 3, Carl Zeiss, Jena, Germany) equipped with a 40X objective lens. The test solutions were applied through a multibarreled pipette positioned close to the target cell. After each experiment on a single cell, the experimental chamber was replaced by another one containing a new sample of cells. All experiments were performed at room temperature (~22 ºC).

The standard tight-seal whole-cell configurations of the patch-clamp technique (Hamill et al., 1981) was used to record macroscopic currents. Glass pipettes were drawn from GB150T-10 glass on a vertical micropipette puller (PUL-100, World precision instruments, Hertfordshire, England) and pipette resistance ranged from 2 to 3 MΩ. Whole-cell currents were filtered with a 4-pole lowpass Bessel filter (Axopatch 200A amplifier) at 2 kHz and digitized (Digidata 1440, Molecular devices) at a sample frequency of 200 kHz (5 µs). The experimental recordings were stored on a computer hard disk for later analysis. Cells were placed in a recording chamber with 0.5 mL extracellular solution containing (in mM): NaCl 50, N-methyl-D-glucamine 90, $CaCl_2$ 2, $MgCl_2$ 1, HEPES 10 and glucose 11; pH was adjusted to 7.4 with HCl. The patch electrodes were filled with pipette solution containing (in mM): CsF 100, CsCl 40, EGTA 10, HEPES 10, NaCl 5, $MgCl_2$ 2, Na_2.ATP 4; the pH was adjusted to 7.3 with CsOH. Once the whole-cell configuration was obtained, current stability was evaluated with a 15 ms-voltage-clamp step from a holding potential of − 80 mV to a test potential of − 20 mV repeated each 10 s. The time needed for the stabilization was variable (approximately 10 min). Series resistance was in the range of 3.5–10 MΩ (recordings with series resistance values exceeding 10 MΩ were excluded from analysis) and was compensated to 60–80%. The same voltage-clamp step protocol was applied in the control (vehicle) or in the presence of compounds $1R^5$, $1R^7$, $3R^5$ and $3R^7$, dissolved in 0.1% dimethylsulfoxide. After current stabilization on each condition, the voltage dependence of the steady-state inactivation of sodium channels was evaluated using a double voltage step protocol, where the same depolarization to − 10 mV followed

different pre-conditioning 2.5 seconds steps (from − 130 to − 40 mV). The available fraction of sodium channels at each membrane potential (IVc/Imax) was calculated as the ratio of peak sodium current measured at −10 mV, at each pre-conditioning voltage test pulse (IVc) and the maximum peak current observed (Imax). The relationship between the available fraction of sodium channels and the pre-conditioning (named h curve) was plotted and fitted with a Boltzmann equation (Eq. (1)): $IVc/Imax = 1/(1 + \exp((Vh - V)/k))$ where the available fraction is given as IVc/Imax, Vh is the potential of half-maximal inactivation and k is the slope parameter. Statistical significance of the changes in the Vh parameter induced by the compounds was tested with F method (GraphPad Prism).
